# Leishmaniasis in Bolivia: Comprehensive Review and Current Status

**DOI:** 10.4269/ajtmh.19-0406a

**Published:** 2019-10

**Authors:** Sergio Mollinedo, Guido Chuquimia, Jhonny Velarde

**Affiliations:** Institute of Health and Environment, La Paz, Bolivia; E-mail: jsergiomollinedo@gmail.com; Health Network No. 3, SEDES La Paz, Bolivia; E-mail: guinochuca6@gmail.com; Epidemiological Surveillance, SEDES Pando, Bolivia; E-mail: gabopando3@gmail.com

Dear Sir,

In the 54th Congress of the Brazilian Society of Tropical Medicine, held in Recife, Brazil, in September 2018, we received several questions regarding the alleged “endemic of visceral leishmaniasis (VL) in the department of Beni (first subnational administrative level) in Bolivia” indicated in the publication of Garcia et al.^1^; unfortunately, this 2009 publication continues to confuse researchers and their current publications.^2^ We have six observations designed to clarify this area. 1) When describing the epidemiology of VL, the authors misstated the initial work of Desjeux et al.,^3^ which clearly indicated that the endemic of VL was in the department of La Paz. 2) The reported data were taken from the official normative documents of the Ministry of Health, written by the persons in charge of the leishmaniasis program (one national level and nine departmental levels)^4^; however, Garcia et al. described the reference as “anonymous.” 3) The report that 31,095 cases of cutaneous leishmaniasis reported in Bolivia between 1983 and 2006 by Garcia et al. is incorrect; the official national document which they referred to indicated 35,714 cases.^4^ 4) Figure 1, which lacked references, was also taken from the official national publication.^4^ 5) Figure 2 similarly had no references; the reported number of cases of cutaneous leishmaniasis was reported in the figure as < 1,000, when in fact 2,043 cases were reported. 6) In referring to “diagnosis,” once again the authors referenced a document as “anonymous,” when in fact, it had an author.^5^
